# Single-Cell RNA Sequencing Analysis of the Early Postnatal Mouse Lens Epithelium

**DOI:** 10.1167/iovs.64.13.37

**Published:** 2023-10-23

**Authors:** Adrienne A. Giannone, Caterina Sellitto, Barbara Rosati, David McKinnon, Thomas W. White

**Affiliations:** 1 Department of Physiology and Biophysics, Stony Brook University School of Medicine, Stony Brook University, Stony Brook, New York, United States; 2 Veterans Affairs Medical Center, Northport, New York, United States; 3 Department of Neurobiology and Behavior, Stony Brook University School of Medicine, Stony Brook University, Stony Brook, New York, United States

**Keywords:** mouse, lens development, single-cell sequencing, postnatal day 2

## Abstract

**Purpose:**

The lens epithelium maintains the overall health of the organ. We used single-cell RNA sequencing (scRNA-seq) technology to assess transcriptional heterogeneity between cells in the postnatal day 2 (P2) epithelium and identify distinct epithelial cell subtypes. Analysis of these data was used to better understand lens growth, differentiation, and homeostasis on P2.

**Methods:**

scRNA-seq on P2 mouse lenses was performed using the 10x Genomics Chromium Single Cell 3′ Kit (v3.1) and short-read Illumina sequencing. Sequence alignment and preprocessing of data were conducted using 10x Genomics Cell Ranger software. Seurat was employed for preprocessing, quality control, dimensionality reduction, and cell clustering, and Monocle was utilized for trajectory analysis to understand the developmental progression of the lens cells. CellChat and GO analyses were used to explore cell–cell communication networks and signaling interactions.

**Results:**

Lens epithelial cells (LECs) were divided into seven subclusters, classified by specific gene markers. The expression of crystallin, cell-cycle, and metabolic genes was not uniform, indicating distinct functional roles of LECs. Trajectory analysis predicted a bifurcation of differentiating and cycling cells from an *Igfbp5+* progenitor pool. We also identified heterogeneity in signaling molecules and pathways, suggesting that cycling and progenitor subclusters have prominent roles in coordinating crosstalk.

**Conclusions:**

scRNA-seq corroborated many known markers of epithelial differentiation and proliferation while providing further insight into the pathways and genes directing these processes. Interestingly, we demonstrated that the developing epithelium can be divided into distinct subpopulations. These clusters reflect the transcriptionally diverse roles of the epithelium in proliferation, signaling, and maintenance.

The anterior surface of the eye lens is covered by a monolayer epithelium that provides the sustenance, coordinated transport, and communication that the lens requires for growth and development and to maintain its optical properties.^1^^,^^2^ In addition, these epithelial cells must differentiate into fibers cells at the lens equator that express high levels of crystallin protein to provide the high refractive index required to focus images on the retina.^3^ During development, epithelial cells undergo proliferation to provide new cells destined for the fiber differentiation pathway and new epithelial cells for the growing surface area of the lens.^4^^,^^5^ Furthermore, the non-proliferating epithelial cells have well established and spatially segregated metabolic and transport functions.^6^^,^^7^ Although derived from a common origin, lens epithelial cells fulfill a variety of specialized functions to maintain the developmental and homeostatic needs of the growing lens.

Lens epithelial cell (LEC) proliferation is dependent on growth factor responses. Specifically, fibroblast growth factor (FGF) induction of mitogen-activated protein kinase/extracellular regulated kinase 1/2 (MAPK/ERK1/2) and the phosphoinositide 3-kinase (PI3K)/Akt signaling pathways have been implicated in epithelial proliferation, migration, and differentiation decisions.^8^^,^^9^ FGF-induced responses in LECs depend on fibroblast growth factor receptors (FGFRs) and subsequent downstream signaling events. In embryonic and early postnatal mouse lenses, LEC proliferation occurs across the anterior epithelium, with the highest levels near the equator.^10^^,^^11^ In older rodent lenses, LEC proliferation is concentrated in a specific area of the epithelial monolayer, located just anterior to the equatorial region, referred to as the germinative zone.^5^^,^^12^

There is a significant increase in LEC proliferation during the first postnatal week of life in mice, which is crucial for the overall growth of the organ. There is a transitory spike in proliferation at postnatal day 2 (P2) followed by a reduction through P7.^10^^,^^11^ This early oscillating rise in cellular proliferation correlates to cell-cycle phases and is important for maintaining the lifelong integrity and structure of the lens.^13^^,^^14^ In order to characterize the different LEC transcriptional states and to probe potential subpopulations that underscore this critical proliferative event, we utilized single-cell RNA sequencing (scRNA-seq) technology. Previous single-cell RNA sequencing studies in the lens have been conducted in zebrafish,^15^^,^^16^ human,^17^^–^^19^ *Drosophila*,^20^ chick,^21^ and adult murine lenses.^22^ However, the early postnatal mouse lens epithelium has not yet been characterized using this method.

Probing the cellular heterogeneity of LECs and understanding the cell–cell communication networks that underlie proliferation, differentiation, and metabolism are critical for understanding overall lens physiology and homeostasis. In this study, we performed scRNA-seq on P2 mouse lenses. Our analysis identified seven subpopulations within P2 LECs that had transcriptionally distinct states. These subpopulations could be further classified into three broader categories: progenitor, cycling, or differentiating cells. Based on known markers of lens differentiation and trajectory analysis, we modeled the differentiation trajectory of LECs and were able to identify the associated genes that were potentially contributing. We also identified differential cell–cell communication networks and signaling pathways engaged in earlier versus later cell states and showed the possible heterogeneity of signaling within LEC subpopulations. This work provides valuable insights into LEC transcriptional organization and lays the foundation for future studies to characterize subpopulations, identify disease states, and interpret key signaling and transcription events in the lens epithelium.

## Methods

### Cell Isolation

Single LECs for RNA sequencing were isolated from P2 wild-type C57BL/6N pups (Taconic Biosciences, Germantown, NY, USA) following an approved Institutional Animal Care and Use Committee protocol and adhering to the ARVO Statement for the Use of Animals in Ophthalmic and Vision Research. Two independent pools of P2 LECs were generated and analyzed. Lenses were dissected from 4 to 8 mouse pups in Tyrode’s solution on a warm stage (37°C). Lenses were first cleaned of the ciliary body and tunica vasculosa using fine forceps, then by digestion in 0.05% trypsin-EDTA for 10 minutes at 37°C and 5% CO_2_. After transfer to ice-cold Tyrode’s solution for 10 minutes, the lens capsule with the adhering epithelial monolayer was peeled off the remaining fiber cell mass using fine forceps and transferred to a tube containing 100 µL of Ca^2+^Mg^2+^-free Dulbecco's phosphate-buffered saline (DPBS; Thermo Fisher Scientific, Waltham, MA, USA) on ice. When all capsules were collected, the tube was moved to 37°C, and 100 µL of 0.1% Collagenase/Dispase Blend (MilliporeSigma, Burlington, MA, USA) was added. After 5 minutes, 1 mL of 0.5% trypsin-EDTA (Thermo Fisher Scientific) was added, and the capsule mass was gently triturated by pipetting, transferred into a culture dish, and trypsinized at 37°C and 5% CO_2_. Trypsinization was monitored under a dissecting microscope until the majority of cells were present as single cells. Minimum Essential Medium (MEM; Thermo Fisher Scientific) supplemented with 10% fetal bovine serum and antibiotics was added, and cells were centrifuged for 5 minutes at 230 relative centrifugal force. The cell pellet was washed once by centrifugation at 4°C in DPBS containing 0.04% RNase-free bovine serum albumin (BSA) (MilliporeSigma). The final pellet was resuspended in the appropriate volume of DPBS/0.04% RNAse-free BSA to have a cell density of 1500 to 2500 cells per microliter and filtered using a 20-µm cell mini-strainer (PluriSelect, Leipzig, Germany). Freshly isolated cell suspensions were immediately processed in the 10x Genomics workflow as described below.

### Single-Cell Sequencing

The scRNA-seq libraries were generated using the Single Cell 3′ Reagent Kit v3.1 (10x Genomics, Pleasanton, CA), according to the manufacturer's instructions. Briefly, cells, gel beads, and partitioning oil were loaded onto a Chromium Next GEM Chip G, for a target recovery of 10,000 cells per sample. The chip was processed in a Chromium Controller (10x Genomics) to generate Gel Beads-in-emulsion (GEMs). After reverse transcription using a GEM–reverse transcription (RT) incubation protocol on a PCR cycler, the GEMs were broken using Recovery Agent (10x Genomics), and the cDNA was purified with DynaBeads MyOne Silane beads (10x Genomics), amplified with PCR for 11 cycles, and further purified with SPRIselect magnetic beads (Beckman Coulter, Brea, CA, USA). Quality control and quantification of recovered PCR products were performed on a TapeStation 4200 with the Agilent High Sensitivity D5000 ScreenTape System (Agilent, Santa Clara, CA, USA). The scRNA-seq libraries were generated using 25% of the cDNA yield (10 µL). After fragmentation, end repair, A-tailing, and size selection purification, the cDNA was ligated with adaptors and purified again with SPRISselect beads. Libraries were amplified using PCR with sample index oligonucleotides from the Chromium i7 Multiplex Kit (10x Genomics), for a total of 10 or 11 PCR cycles, as estimated from the initial cDNA input. The final PCR products were subjected to double-sided size selection with SPRISselect beads (0.6× and 0.8×). Library quality control was performed on the Agilent TapeStation 4200, and the library yield was quantified by quantitative PCR (qPCR) using the KAPA Library Quantification Kit (Roche Diagnostics, Indianapolis, IN, USA). Sequencing was performed through a commercial supplier (Novogene, Durham, NC, USA) at a depth of 20,000 paired reads per cell on an Illumina NovaSeq 6000 sequencer with the following (PE150) sequencing settings: Read 1, 151 cycles; i7 index, 8 cycles; Read 2, 151 cycles.

### Preprocessing, Quality Control, Clustering, Integration, and Cell-Type Identification

Raw data were aligned to the mouse genome (mm10) reference transcriptome and underwent preprocessing with Cell Ranger 6.1. The count matrix was then analyzed using Seurat 4.3.0 (R Foundation for Statistical Computing, Vienna, Austria).^23^ Filtering included removal of cells in the bottom 2% and upper 3% of expressed genes (nGene) and expressed RNA (nUMI) fractions and removal of cells with more than 15% mitochondrial reads. Approximately 8% of the droplets were identified as containing multipets by DoubletFinder^24^ and were removed from further analysis (Supplementary Figs. S1, S2).

Normalization was performed using SCTransform followed by regression of cycle genes. The two independent samples were integrated with Harmony.^25^ Dimensionality reduction was performed using principal component analysis and Uniform Manifold Approximation and Projection (UMAP). Clustering was performed using the Louvain algorithm. Canonical gene markers were used to identify four unique cell types within 11 cell subpopulations.

After this initial analysis, the LECs were identified and subset for further analysis. Gene set enrichment was performed using the clusterProfiler and Fast Gene Set Enrichment Analysis (fgsea) R packages. The results of the Gene Ontology (GO), Reactome, and Hallmark pathway analysis were filtered using a Benjamini–Hochberg (BH)-adjusted *P* < 0.05 and sorted by normalized enrichment score (NES) magnitude.

### Cell Trajectory and Pseudotime Analysis

Pseudotime modeling was performed using Monocle 3 (v1.3.1).^26^ Trajectories were identified and cells ordered based on pseudotime using the learn_graph function and visualized using the plot_cells function, respectively. The Progenitor cluster (cluster 0) was used as the root cluster. For downstream analysis, estimate_size_factors was first used to normalize gene expression, and then graph_test was run to look at differential gene expression throughout pseudotime. Gene modules were then identified using the find_gene_modules function, which utilizes the output of the graph_test function to group genes together based on their variance patterns along pseudotime. Gene outputs from the top two gene modules for each Seurat cluster were then classified using GO pathway analysis and submitted to PantherDB.org.^27^

### Cell Communication Analysis

In order to quantify and visualize cell signaling and communication networks within LECs, CellChat 1.6.1 was utilized using the standard pipeline.^28^ The entire CellChat database was used to analyze known ligand–receptor interactions between LECs and identify signaling pathways that may be present in LEC subpopulations.

## Results

### Identification of Cell Types in the P2 Mouse Lens

**Figure 1.**
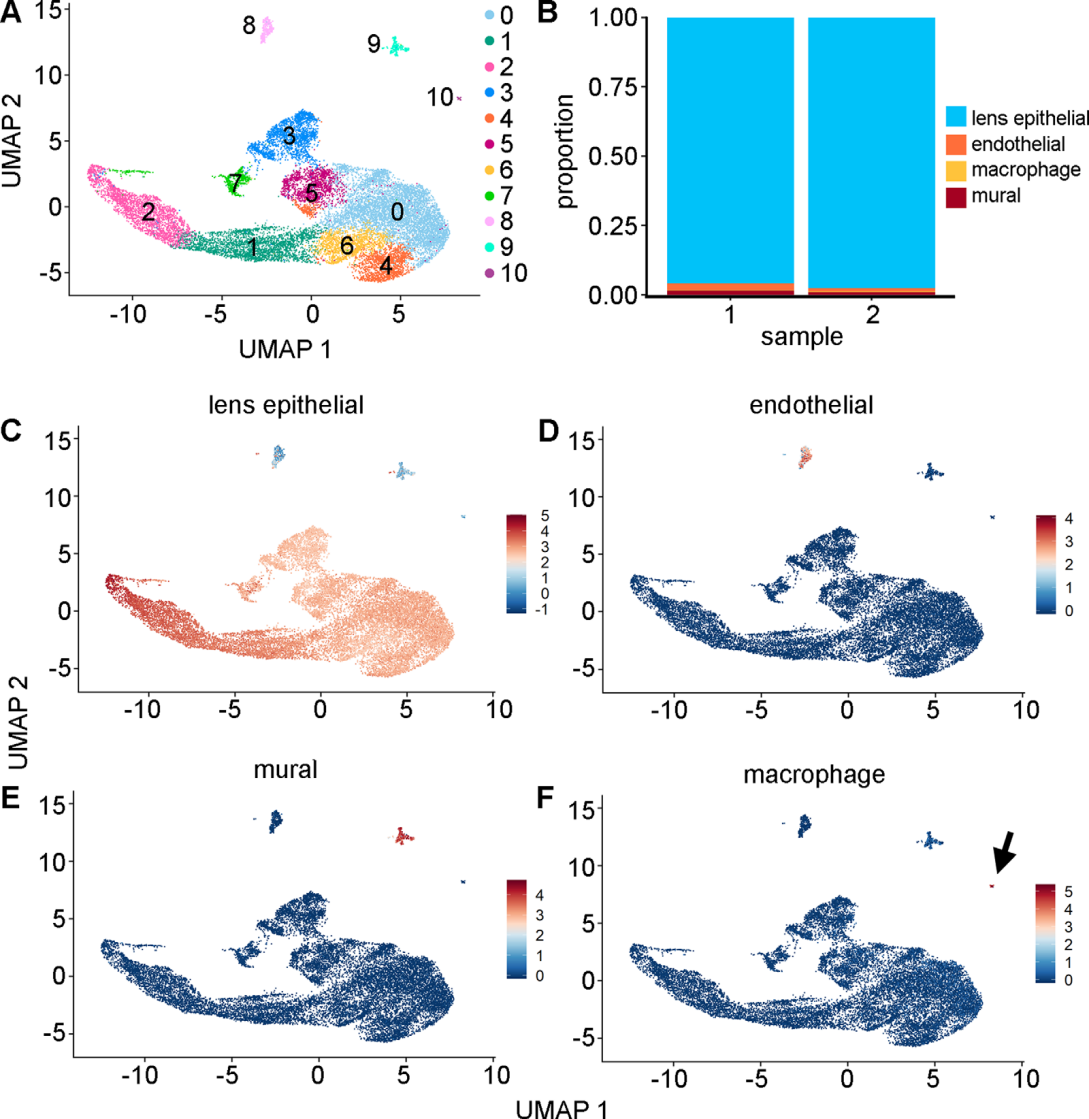
(**A**) In this study, 18,776 post-filtered cells were combined from two independent biological samples and visualized using a UMAP representation. To allow initial assessment of biological variation, the number of genes, number of transcripts, and mitochondrial and ribosomal RNA counts were not regressed out as sources of variation. In this analysis, the Louvain algorithm (resolution = 0.3) identified 11 clusters. (**B**) Proportion of cell-type numbers based on identified cell types for each of the two samples. Cell types were identified based on average expression of canonical gene markers for the four main cell types: (**C**) lens epithelial cells (*Cryaa*, *Cryab*, *Gja8*); (**D**) endothelial cells (*Cldn5*, *Col18a1*, *Ctla2a*); (**E**) mural cells (*Rgs5*, *Mgp*, *Serpine2*); and (**F**) macrophages (*Pf4*, *C1qa*, *Apoe*). The macrophage cluster is indicated by an *arrow*.

Two independent samples of freshly isolated P2 LECs were obtained from wild-type mice and analyzed using scRNA-seq. Cell types were manually identified based on the expression of canonical gene markers. This analysis suggested the presence of eight contiguous clusters of lens epithelial cells (clusters 0–7), and three single small clusters of endothelial cells (cluster 8), mural cells (cluster 9), and macrophages (cluster 10) (Fig. 1A). The majority (96.6%) of filtered cells were determined to be LECs (Fig. 1B). LEC identity (Fig. 1C) was inferred from high expression of known marker genes for this cell type, including the abundant crystallins *Cryaa* (αA crystallin) and *Cryab* (αB crystallin),^29^ as well as *Gja8* (connexin 50).^30^^–^^34^ Endothelial cells (Fig. 1D) were characterized by *Cldn5* and *Ctla2a* expression. *Cldn5* is abundantly expressed in microvascular endothelial cells, and *Ctla2a* is a capillary endothelial marker.^35^^,^^36^ Mural cells (Fig. 1E) were categorized by expression of the marker genes *Rgs5* and *Serpine2*, which are vascular maturation and calcification factors, respectively.^37^^,^^38^ Macrophages (Fig. 1F) were detected by expression of the complement system gene *C1qa*, as well as *Apoe.*^39^^,^^40^ Within the identified LEC groups, cluster 7 was anomalous based on low median genes per cell, low median unique molecular identifier (UMI) counts per cell, a low percentage of mitochondrial RNAs, and a high percentage of ribosomal RNAs (Supplementary Fig. S2). These cells were assumed to be damaged, or stressed, and were removed from further downstream analysis.

### LEC Subtypes Displayed Differential Gene Expression

**Figure 2.**
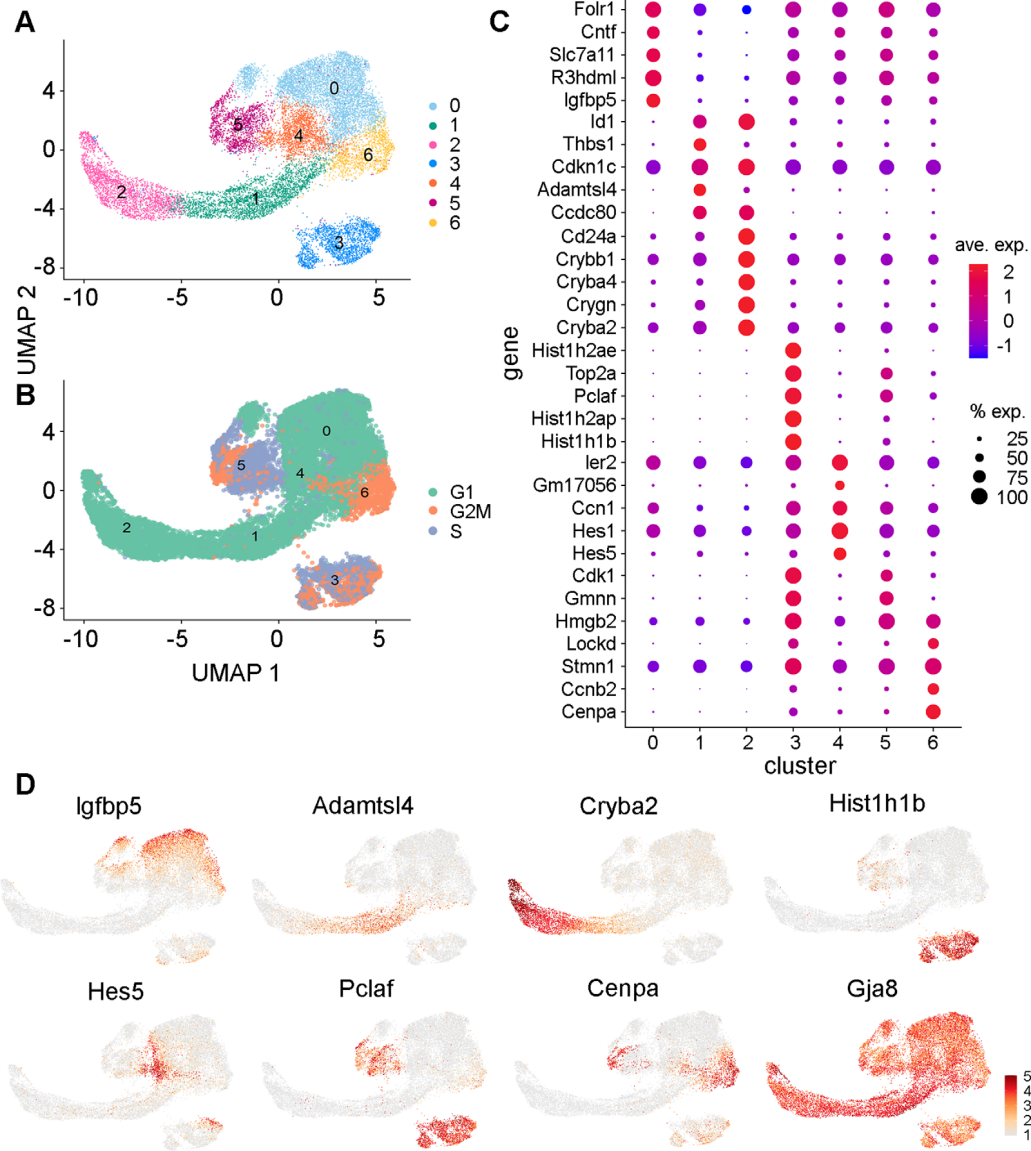
(**A**) UMAP representation of LECs after removal of other cell types and damaged cells. Lovain clustering (resolution, 0.2) identified seven subtypes. (**B**) Cell-cycle scores for each cluster. (**C**) Dot plot showing the top five differentially expressed genes for each of the seven LEC clusters. Duplicated markers are only shown once. (**D**) Examples of patterns of cluster-specific gene expression for seven selected marker genes and one ubiquitously expressed gene (*Gja8*).

**Figure 3.**
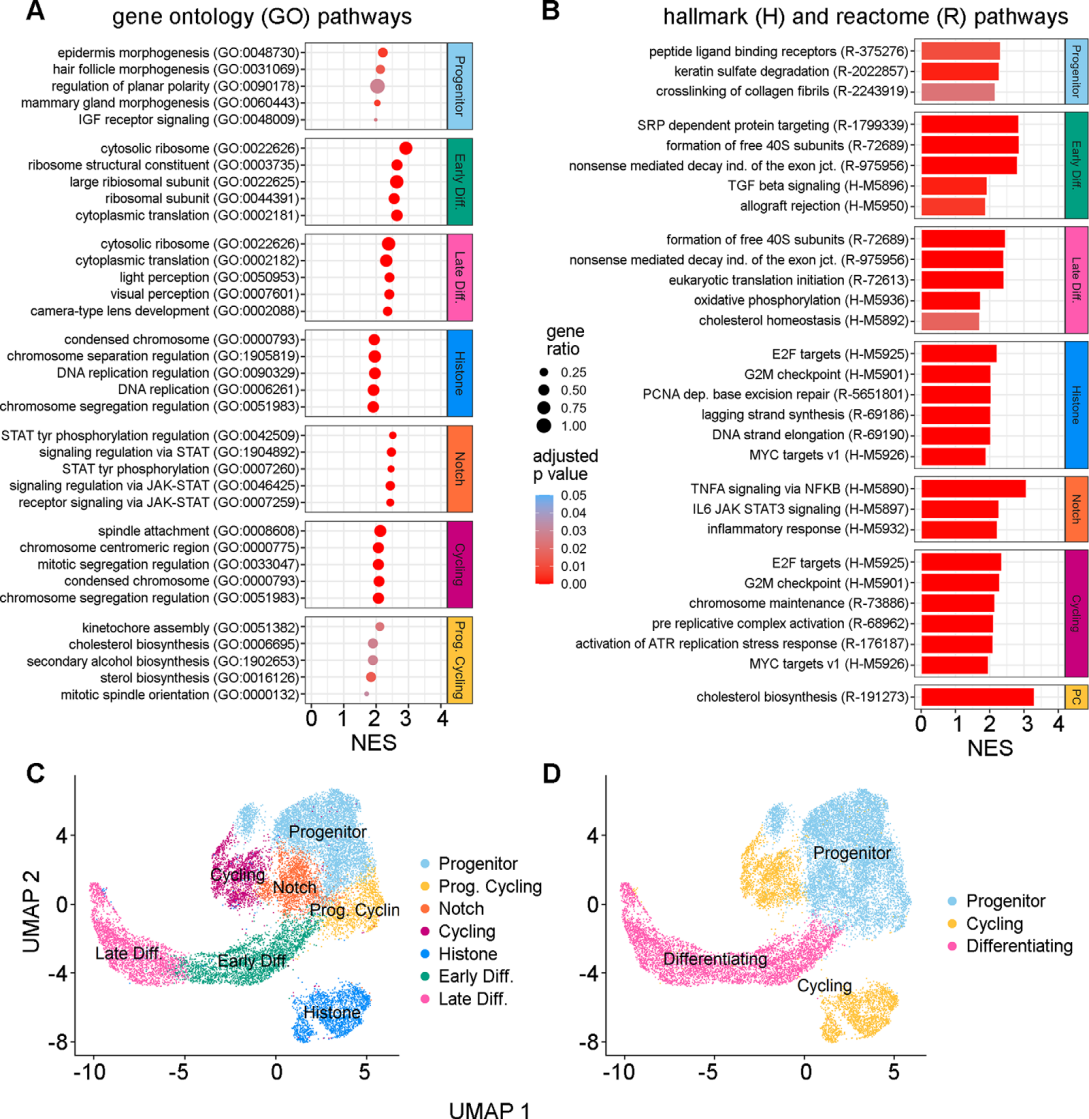
(**A**) GO term enrichment analysis. Only the top (*n* = 5) significant (adjusted *P* < 0.05) and upregulated (NES > 0) annotations per cluster are shown and ordered by NES magnitude. *Dot color* represents the adjusted *P* value intensity, and the *dot size* represents the gene ratio. (**B**) Reactome and Hallmark pathway enrichment. The top significant (adjusted *P* < 0.05) Reactome (*n* = 3) and Hallmark (*n* = 3) pathways are shown and ordered by NES magnitude. Some clusters did not have multiple significant pathways. *Scale bar intensity* reflects the adjusted *P* value. (**C**) Seven identified clusters are shown in a UMAP representation. (**D**) Three higher level groups are shown in a UMAP representation.

Following annotation of cell types within the complete sample, LECs were subset and re-clustered (Fig. 2A). Approximately 33% of LECs were scored as cycling, with 14% in the G2M phase and 19% in the S phase (Fig. 2B). Among the seven identified LEC clusters remaining after subsetting, there was considerable heterogeneity of gene expression (Fig. 2C). In cluster 0, there was high expression of nutrient-responsive genes, such as the insulin-like growth factor (IGF) binding protein *Igfbp5*, the nutrient binding proteins *Slc7a11* and *Folr1*, and the neurogenic factor *Cntf* (Figs. 2C, 2D). *Igfbp5* is an IGF binding factor that plays a role in insulin signaling and early postnatal lens development and is regulated by *Pax6.*^41^ IGF signaling in the lens also mediates proliferation, differentiation, and migration decisions during development.^42^ *Slc7a11* is an amino acid transporter that has roles in growth and metabolism and is present in both LECs and lens fiber cells (LFCs) in zebrafish.^15^ *Slc7a11* is a cystine/glutamate transporter that is involved in redox homeostasis by regulating intracellular glutathione (GSH) levels.^43^ GSH is an essential antioxidant in the lens for maintaining optical lens clarity.^44^ *Folr1* is a folate receptor that mediates the uptake of folate from the bloodstream, which is crucial for proper lens development.^45^ GO analysis was used to functionally annotate the genes upregulated in cluster 0. This analysis showed that these genes had roles in cellular morphogenesis, epithelial maturation, collagen secretion, and IGF signaling (Fig. 3). This analysis identified several other signal pathway genes upregulated in cluster 0, including the bone morphogenetic protein (BMP) antagonist *Sostdc1*, the epithelial cell development factor *Klf4*, the cysteine protease *Ctsl*, the Wnt co-receptor *Fzd2*, *Tfap2a* (AP-2a), and the transforming growth factor (TGF) signaling factor *Tgfb2*. Specific collagen genes, such as *Pcolce*, were also upregulated in this cluster. Given the established roles of these differentially expressed genes, as well as the predominant G1 cell cycle state, this cluster was labeled the “progenitor” cluster.

In contrast, cell cluster 1 expressed many genes important for lens biology, such as *Cdkn1c*, *Ccdc80*, and *Adamtsl4*. *Cdkn1c* (also known as *p57^Kip2^*) expression is a key marker of LEC cell cycle exit and initiation into differentiation.^46^ The gene *Ccdc80* has strong homology to equarin, which is also a mediator of LEC differentiation in chick embryos.^47^ *Thbs1* (thrombospondin) and *Id1* are both involved in mediating TGF-β signaling and activation in the lens.^48^^,^^49^ *Adamtsl4* has a proposed role of anchoring zonular fibers to the lens equatorial region.^50^ Enriched GO terms in this cluster included genes mainly involved in ribosome biosynthesis and translation. An increase in ribosomal production could indicate translation of genes necessary for differentiation, especially given the co-expression of *Cdkn1c* in this cluster.^51^ Ribosomal RNA production is highly enriched at the lens transitional zone.^52^ Differentially expressed genes identified by GO terms included *Rbm24*, which plays a role in crystallin mRNA translation and LEC terminal differentiation, and the laminin gene *Lama2.*^53^ Given the expression of genes involved in cell-cycle exit and initiation into differentiation, this cluster was identified as the “early differentiation” cluster.

Cluster 2 displayed abundant expression of beta and gamma crystallin genes, such as *Cryba2*, indicating that this cluster may contain cells further along the differentiation pathway than cluster 1.^54^ Additionally, there was marked expression of *Cd24a*, which is a cell membrane adhesion protein known to be highly expressed in LFCs.^55^ Expression of gamma crystallins starts to accumulate during epithelial elongation in cells located posteriorly to the equatorial region, as does *Crybb1.*^56^ Crystallin expression may be temporally distinct, with gamma and beta crystallins specifically expressed later in the process of differentiation.^57^ Additional cluster 2 genes associated with GO terms were mainly crystallin genes, but we also identified an increase in *Aqp0*/*Mip* (major intrinsic protein) expression in this cluster, as well as *Maf* (c-Maf), *Nhs*, *Bfsp1* (filensin), and *Lim2* (lens intrinsic membrane protein 2). The cells in this cluster had upregulation of genes related to fiber cell differentiation; therefore, this cluster was labeled the “late differentiating” cluster.

Cells in cluster 3 had cell-cycle scores predominantly in the G2/M and S phases and had distinct and unique expression of many histone-related genes, such as *Hist1h1b*, *Hist1h2ap*, and *Hist1a2ae*. Histone genes are transcribed throughout the cell cycle, with a large increase in histone mRNA levels during the S phase followed by selective degradation at the completion of DNA replication.^58^ Histone posttranslational modifications and chromatin remodeling have important roles in lens development and LEC differentiation.^59^^,^^60^ Mutations in histone genes, such as *Hist2h3c1*, have also been linked to cataract formation and an observed decrease in crystallin and *Aqp0* expression during development.^61^ In crystallin-deficient lenses, the relative ratios of histones H2A/H2B and H3/H4 are altered, suggesting a synergistic relationship between crystallins and histones.^62^ However, the relative abundance and roles of these histones in LEC subpopulations have yet to be established. Cluster 3 also displayed expression of *Pclaf*, which plays an essential role in cell proliferation but has not yet been described in the lens.^63^ The GO analysis primarily identified an increase in cell cycle genes. Other differentially expressed genes identified by the GO analysis included the cell-cycle regulators *Fbxo5* and *Dbf4*. Based on these observations, cluster 3 was labeled the “Histone” cluster.

For cells in cluster 4, there was upregulated expression of the Notch effectors *Hes5* and *Hes1*. Notch signaling directly suppresses *Cdkn1c* in the lens epithelium and maintains a population of proliferating LECs.^46^ During development, Notch effectors and recipients in LECs have spatially distinct expression that helps coordinate the decision to differentiate or proliferate.^64^ The GO analysis identified that genetic markers of this cluster may be involved in Janus kinase (JAK)/signal transducer and activator of transcription (STAT) and tumor necrosis factor A (TNFA)/nuclear factor kappa B (NF-κB) signaling. Differentially expressed genes identified by GO analysis included *Socs3*, *Junb*, *Zfp36*, and *Fos*/*Jun* (AP-1). Due to the distinctive *Hes5*/*Hes1* expression, cluster 4 was labeled the “Notch Effector” cluster.

Cluster 5 contained cells in the G2M and S phases of the cell cycle and expressed several markers of cycling cells. This cluster had marked expression of *Cdk1* and *Gmnn*. *Cdk1* is an essential cell-cycle gene that controls progression through mitosis and is rarely expressed in post-mitotic cells.^65^ *Gmnn* (geminin) is an S-phase regulator that also associates with chromatin.^66^ Cluster 5 also had elevated expression of *Pclaf*, which is a cell-cycle regulator.^63^ As these genes are key for cell-cycle progression, this cluster was labeled the “Cycling” cluster.

Cluster 6 contained cells primarily in the G2M phase that expressed genes more similar to the Progenitor cluster cell population than the other cycling clusters. Unique markers of this cluster included *Ccnb2* and *Cenpa*. *Ccnb2* (cyclin B2) has known function in LEC mitosis and is also expressed during terminal fiber cell differentiation.^67^ *Cenpa* is a structural centromeric protein that also functions in nucleosome formation and regulation of chromatin.^68^ GO analysis uniquely identified an upregulation of sterol and alcohol biosynthesis in this cluster, indicating that metabolic pathways may be present. Some genes identified from the GO analysis were *Fdps*, *Hmgcs1*, and *Msmo1*. These genes have been previously identified in the lens as regulators of cholesterol biosynthesis.^69^ Interestingly, these biosynthetic genes were also upregulated in the Late Differentiating cluster. Given the similarities to progenitor cells and their biosynthetic properties, this cluster was labeled “Progenitor Cycling.”

The relationship among these seven identified gene clusters was plotted in a UMAP representation (Fig. 3C). Based on hierarchical analysis of gene expression patterns, as well as the analysis of gene modules described below using Monocle (Supplementary Figs. S3B, S3C), it appeared likely that these clusters could also be grouped into three main classes: progenitor cells, cycling cells, and differentiating cells (Fig. 3D).

### Inferred Pseudotime Trajectories Supported Gene Expression–Based Cell Group Assignments

**Figure 4.**
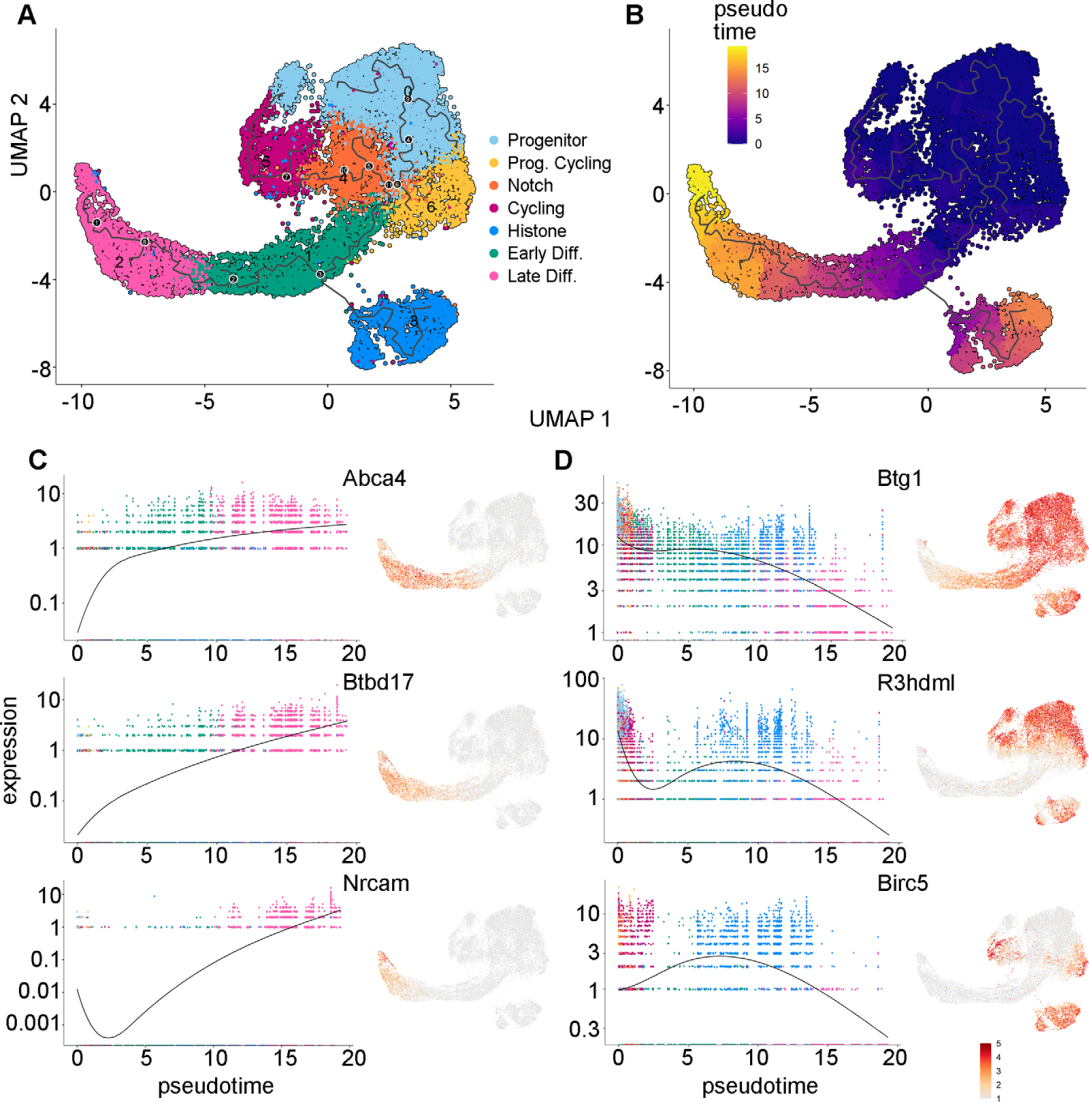
(**A**) The differentiation trajectory for lens epithelial cells inferred by Monocle 3, with labeled branch points (*black dots*). (**B**) UMAP representation colored by Monocle pseudotime scores. *Purple* represents cells that are earlier in pseudotime, and *yellow* indicates cells that are later in pseudotime. (**C**, **D**) For selected genes, a paired single-cell expression versus pseudotime plot (*left*) and a UMAP representation colored with relative gene expression (*right*) are shown. Plots of genes determined to be expressed later in pseudotime (**C**) and earlier in pseudotime (**D**) are shown.

Monocle 3 was used to infer cell state changes as a function of progress along a trajectory termed “pseudotime.” Pseudotime analysis confirmed the direction of differentiation states among the identified cell clusters. Monocle uses known genetic networks involved in proliferation and differentiation to unbiasedly assume temporal states of cells.^26^ Starting from the Progenitor cluster, the pseudotime analysis suggested an ordering of the differentiation progression through the different cell groups to end in the Late Differentiation cluster (Figs. 4A, 4B; Supplementary Fig. S3A). Monocle also identified genes that varied or changed as a function of pseudotime. Among the genes that increased with pseudotime were *Abca4*, *Btbd17*, and *Nrcam* (Fig. 4C). The shape and attachments of LECs change throughout the process of differentiation, and this process may be aided by *Nrcam* and another late expression gene, *Pmp22*, which are both neuronal cell adhesion genes.^70^ *Abca4* is also an important membrane-associated protein involved in retinal metabolism.^71^ Relatively little is known about *Btbd17*, but it is also expressed in the brain and is predicted to localize in the plasma membrane.^72^ Genes expressed early in pseudotime that declined in expression during development included *Btg1*, *R3hdml*, and *Birc5* (survivin) (Fig. 4D). *Btg1* is an antiproliferation factor associated with cellular differentiation and is known to be expressed in chick lens vesicles during development.^73^ *R3hdml* is a TGF-β–associated signaling protein, but its specific function in the lens remains unknown.^74^ *Birc5* (survivin) expression is correlated with proliferating cell subpopulations in chick lenses and is downregulated in differentiating LECs.^75^ The top two expressed modules per cluster were analyzed using PantherDB GO analysis, which further functionally annotated the cell subpopulations (Supplementary Fig. S3B, Supplementary Table S1). A complete list of genes that were determined to be expressed in specific clusters is provided in Supplementary Table S2.

### CellChat Identified Potential Signaling in the Different LEC Subpopulations

**Figure 5.**
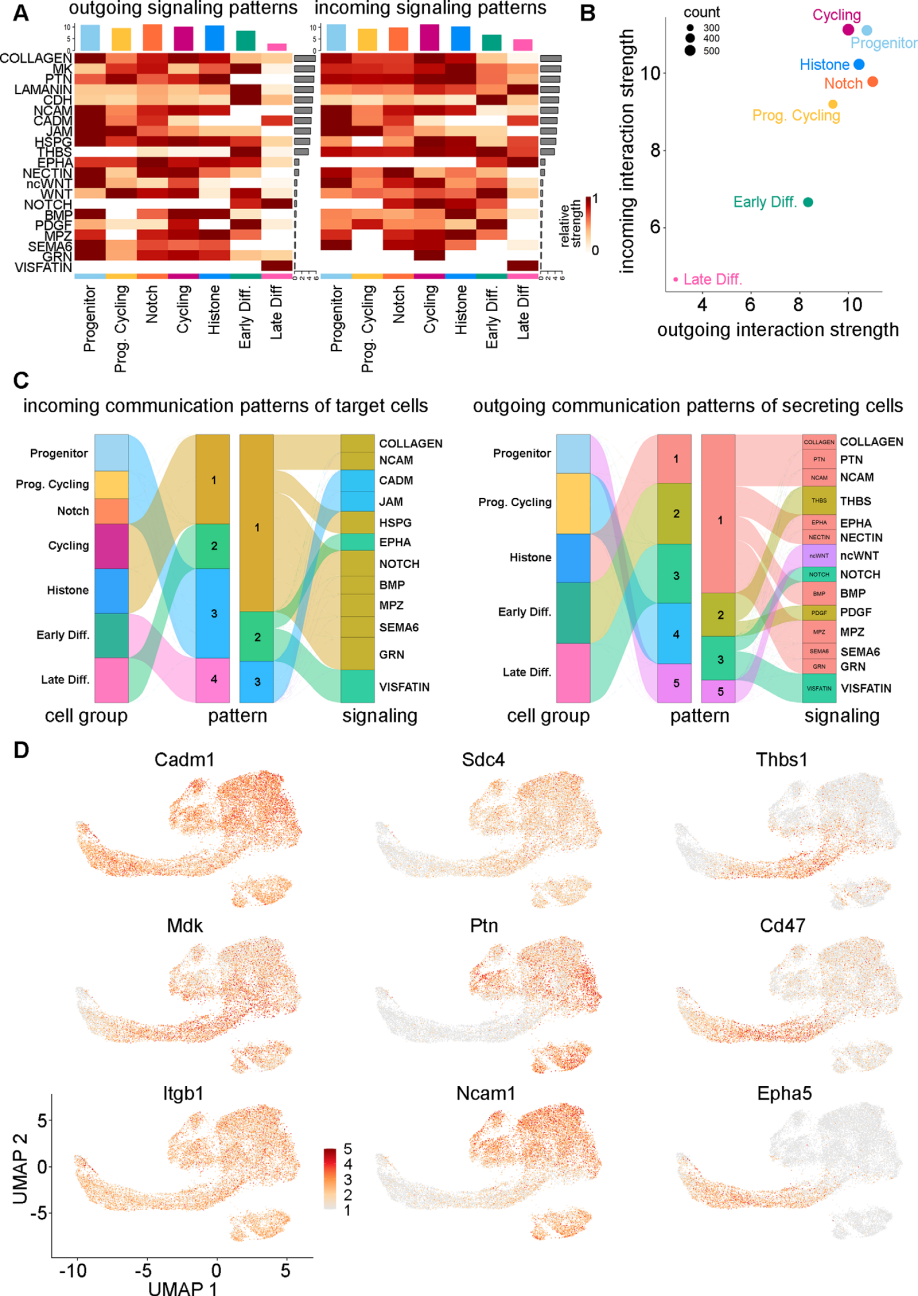
(**A**) CellChat net analysis heatmap of both outgoing (*left*) and incoming (*right*) significant signaling pathways per LEC subtype. Pathways are ordered by the relative strength of receptor–ligand expression and signaling communication pathway probability. (**B**) CellChat net analysis scatterplot showing the overall interaction strength of each LEC subtype to both outgoing and incoming signal strength. (**C**) River plots showing incoming (*left*) and outgoing (*right*) signaling patterns from LEC subtypes and the pathways that comprise each type of identified pattern. (**D**) UMAP representation of the expression of selected signaling molecule identified through CellChat analysis.

Signaling networks that might be active in the different LEC subpopulations were inferred and analyzed using CellChat.^28^ A general pattern that emerged was a reduction in the intensity of signaling in cell clusters identified as being in later states of differentiation (Figs. 5A, 5B), consistent with the approach toward the more inert fiber cell state. The networks that appeared to make the strongest contributions to signaling in LECs were the collagen, midkine (MK), and pleiotrophin (PTN) signaling networks (Fig. 5A, Supplementary Fig. S4). The collagen signaling network was expressed in all clusters, but there was some differentiation of collagen expression; for example, *Col6a3* expression was upregulated in the Late Differentiating cluster (Supplementary Fig. S4C). The heparin-binding growth factors midkine (*Mdk*) and pleiotrophin (*Ptn*) were downregulated in the Late Differentiating cluster. The downstream signaling from MK and PTN receptor binding includes ERK and PI3K in other cell systems and is involved in epithelial to mesenchymal transition interactions and migration during development.^76^^,^^77^ *Mdk* expression was ubiquitous in LECs, whereas *Ptn* expression disappeared in both the Early and Late Differentiating clusters (Supplementary Fig. S4C). The signaling pathways upregulated in the progenitor and cell-cycling clusters mainly included those involved in extracellular matrix (ECM) secretions, such as neural cell adhesion molecules (NCAMs), heparin sulfate proteoglycans (HSPGs), pleiotrophin, and junctional adhesion molecules (JAMs) (Fig. 5C). Pathways that were more prevalent in differentiating clusters included laminin, cadherin (CDH), thrombospondin (THBS), platelet-derived growth factor (PDGF), Notch, and visfatin. Some signaling genes, such as *Cadm1*, *Mdk*, and *Itgb1*, are expressed relatively ubiquitously in LECs, whereas other genes may be expressed uniquely during differentiation, such as *Cd47* and *Epha5* (Fig. 5D, Supplementary Fig. S4). Signaling genes upregulated earlier in pseudotime included *Ptn*, *Ncam1*, and *Sdc4*.

## Discussion

The postnatal murine lens epithelium contains a pool of progenitor cells, which not only are the source of future fiber cells but also differentiate into diverse epithelial cell subtypes that are responsible for the nourishment, solute transport, and communication that the lens requires for normal growth and development.^78^^,^^79^ In this study, we used scRNA-seq to provide an in-depth transcriptomic analysis of single LECs in order to probe the transcriptional and functional diversity of LEC subtypes in the early postnatal period. The data we obtained provided a snapshot of mouse lens growth and development on P2. We found that there were three major subtypes of lens epithelial cells in the mouse lens at P2: progenitor, cycling, and differentiating. These subtypes were further subdivided into seven distinct clusters based on different patterns of gene expression. The small fraction of contaminating cell types was likely derived from the tunica vasculosa lentis (TVL). Previous studies have identified the presence of mural, macrophage, and vascular endothelial cells in the TVL during this period of lens growth.^80^^–^^82^

The progenitor cell clusters displayed increased metabolic functions, including GSH synthesis pathways and lipid metabolism and synthesis. This correlates with a previous rodent lens study that identified a large increase in lipid synthesis during the first postnatal weeks.^83^ Therefore, these two subpopulations of LECs could have specialized functions in maintaining the intense metabolic state during the first postnatal week of life. Interestingly, the Progenitor Cycling cluster may also be involved in progenitor self-renewal and growth of the lens during this period. Cell-cycle scoring predicted that P2 LECs had approximately a third (33.35%) of the cells undergoing the cell cycle, with approximately 20% (19.14%) in S phase, which correlates exceptionally well with analysis based on BrdU staining at P2.^10^ Future studies could use scRNA-seq at other postnatal ages to track if cycling cell number decreases by P6, as was also previously documented.^10^^,^^11^ Changes in the proportion of the different cell types would also be expected, because during P2 there are highly proliferative cells in both the anterior and equatorial regions, whereas by P14 these cells are almost solely concentrated in the germinative zone.^84^^,^^85^

Spatiotemporal organization of LEC subclusters could be inferred from known cell markers, as well as pseudotime analysis. Notably, high expression of *c-Maf*, *Jag1*, *Prox1*, and *Cdkn1c* (p57/KIP2) was shown throughout the early and late differentiating clusters (Supplementary Fig. S5), suggesting localization toward the lens equator. These genes are known regulators of LEC cycle exit and subsequent fiber cell differentiation.^86^^–^^88^ Earlier in pseudotime, there was increased expression of *Mafb*, *Itga3*, *Cdh1*, and *Bmp7*, which are genes with established roles in lens epithelial homeostasis and development^89^^–^^91^ (Supplementary Fig. S5). The upregulation of biosynthetic genes in both the Progenitor and Progenitor Cycling cluster also suggests potential functional similarities among these cell populations. Upregulation of cell-cycle genes, such as *Cdk1*, and the Seurat cell cycle scoring function were able to identify cell subpopulations undergoing the cell cycle.^67^^,^^92^

On P2, cell division in the mouse lens extends across the entire epithelium.^10^^,^^11^^,^^85^ Thus, it could be hypothesized that the Progenitor and Progenitor Cycling clusters may occupy much of the anterior central zone, the Notch effector cluster could be closer to the germinative zone, and the Histone and Cycling clusters are likely anterior as well as within the germinative zone. The Early and Late Differentiating Clusters could be at the equatorial and transitional zones. Future experiments such as in situ hybridization, immunofluorescence, and spatial transcriptomics should be conducted to provide more definitive insights into the precise spatial localization of these clusters based on identified differentially expressed genes (DEGs).

Regarding cell signaling, we found that the top expressed receptor–ligand interactions originated from the collagen, midkine, and pleiotrophin pathways. The Progenitor and Cycling clusters had the most extensive signaling interactions, whereas the Differentiating clusters comparatively had comparatively lower signaling interactions. Notably, we observed heterogeneous expression of signaling molecules in cells earlier versus later in pseudotime, with some specific to cells assumed to be undergoing differentiation, including the THBS and Notch pathways. Notch signaling is known to be important in mediating epithelial to fiber cell differentiation^88^ and was identified by CellChat to be upregulated in the early and late differentiating clusters only (Fig. 5). Concurrently, signaling molecules such as thrombospondin-1 also showed downregulation in the late differentiating cells relative to the early differentiating cells, which coincides with a previous study.^49^ The high expression of the collagen, laminin, CDH, NCAM, cell adhesion molecule (CADM), and HSPG signaling networks is likely due to coordination of lens capsule formation or ECM remodeling and cellular migration during differentiation events. For example, the laminin and CDH networks were more prevalent in differentiating cells, whereas NCAM, CADM, JAM, HSPG, and collagen signaling events were more prevalent in progenitor and cycling cells. In addition, the gene *Itga6* (integrin subunit alpha 6) in the MK signaling system was expressed higher in the Late Differentiating cluster (Supplementary Fig. S4), which correlates with a prior in vitro study in rats.^93^ In contrast, signaling molecules such as *Ptn* were not expressed at all in the Differentiating clusters (Supplementary Fig. S4). Other signaling pathways, such as the noncanonical Wnt (ncWnt), Wnt, BMP, and myelin protein zero (MPZ), had variable signaling patterns but were mainly downregulated in the differentiating clusters. All of these pathways have established roles in lens development.^94^^–^^96^ Understanding the heterogeneity of signaling in the lens epithelium is crucial for identifying how lens formation and cellular organization occur.

Some of the LEC clusters identified in our study could have distinct functions in transitioning between the proliferative and differentiating state. The Notch Effector cluster, which was also identified by pseudotime, could contain specialized cells that coordinate the differentiation versus proliferation decision.^88^ In the Differentiating cell clusters, there was also an increase in ribosomal protein GO terms and expression, including *Rbm24*, which is specific to crystallin synthesis. Recently, the role of ribosomal proteins in lens translational control during development has been studied in depth, and specific translation control was found to be crucial for regulating LECs differentiation and specific protein expression.^51^ In contrast, the prominent cycling histone expressing cluster remains elusive in its function. It is possible that histone remodeling could indicate a commitment to differentiation,^97^^,^^98^ which is consistent with the modeling that placed this cluster later in pseudotime (Fig. 4). Alternatively, it may be linked to the transient state of high proliferation during the first postnatal week.^10^ Future studies looking at histone proteins in the lens, as well as other postnatal time points, may provide insights into their functions and potential roles in differentiation commitment.

Single-cell RNA sequencing has allowed the cellular heterogeneity of postnatal LECs to be explored and has highlighted cell–cell communication networks that underlie proliferation, differentiation, and metabolism. Our findings are largely consistent with prior studies of adult human and mouse LECs. Previous scRNA-seq of human lens tissue also identified similar LEC subpopulations,^19^ as did a study of adult mouse LECs.^99^ This study has defined a transcriptomic atlas for the P2 mouse lens epithelium and cataloged the differential gene expression patterns of LEC subpopulations, suggesting a lineage differentiation trajectory for them. Our findings indicate that LEC heterogeneity in the developing lens is key for coordinating metabolism, self-renewal, and differentiation.

## Supplementary Material

Supplement 1

Supplement 2

Supplement 3
