# Supplementary material for: Single-Cell RNA Sequencing Analysis of the Early Postnatal Mouse Lens Epithelium
Source: Invest Ophthalmol Vis Sci. 2023 Oct 23;64(13):37. doi: 10.1167/iovs.64.13.37 (PMC10599162; doi:10.1167/iovs.64.13.37)
Supplement: Supplement 1 [file iovs-64-13-37_s001.pdf]

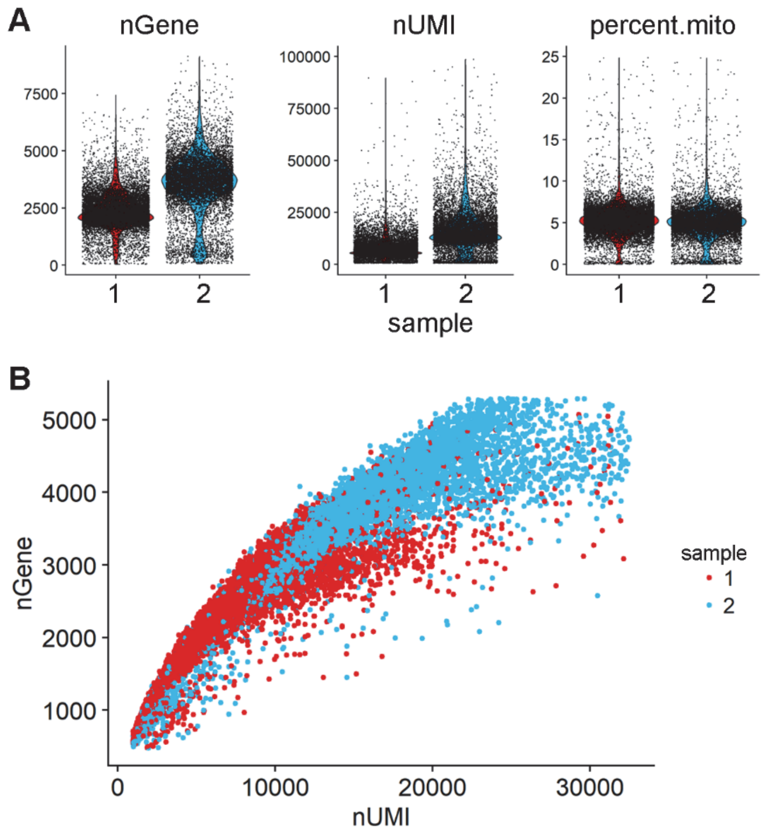

Supplementary Figure 1. (A) Violin Plots showing the distribution of unfiltered data for the number of genes per cell, number of UMIs per cell, and percent mitochondrial genes expressed. (B) Scatter plot showing the degree of correlation between the number of UMIs per cell and number of genes per cell after filtering.

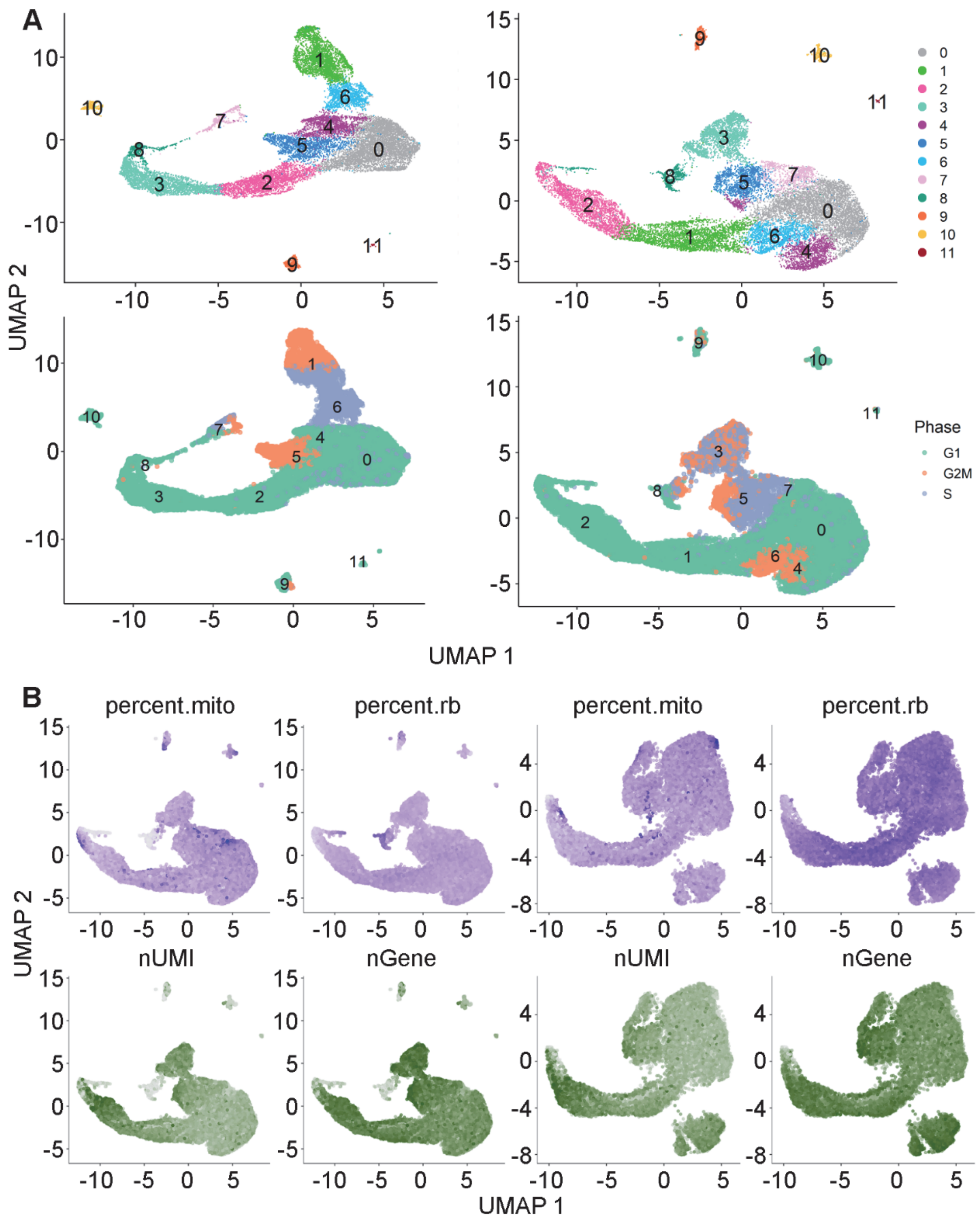

Supplementary Figure 2. (A) UMAP representation of the entire dataset (left) and the same dataset after regression on cell cycle (right). (B) UMAP representation of percent mitochondrial gene expression, percent ribosomal gene expression, total UMI expression per cell and total genes per cell. All cells shown in left-hand panel and the LEC subclusters shown in the right-hand panel.

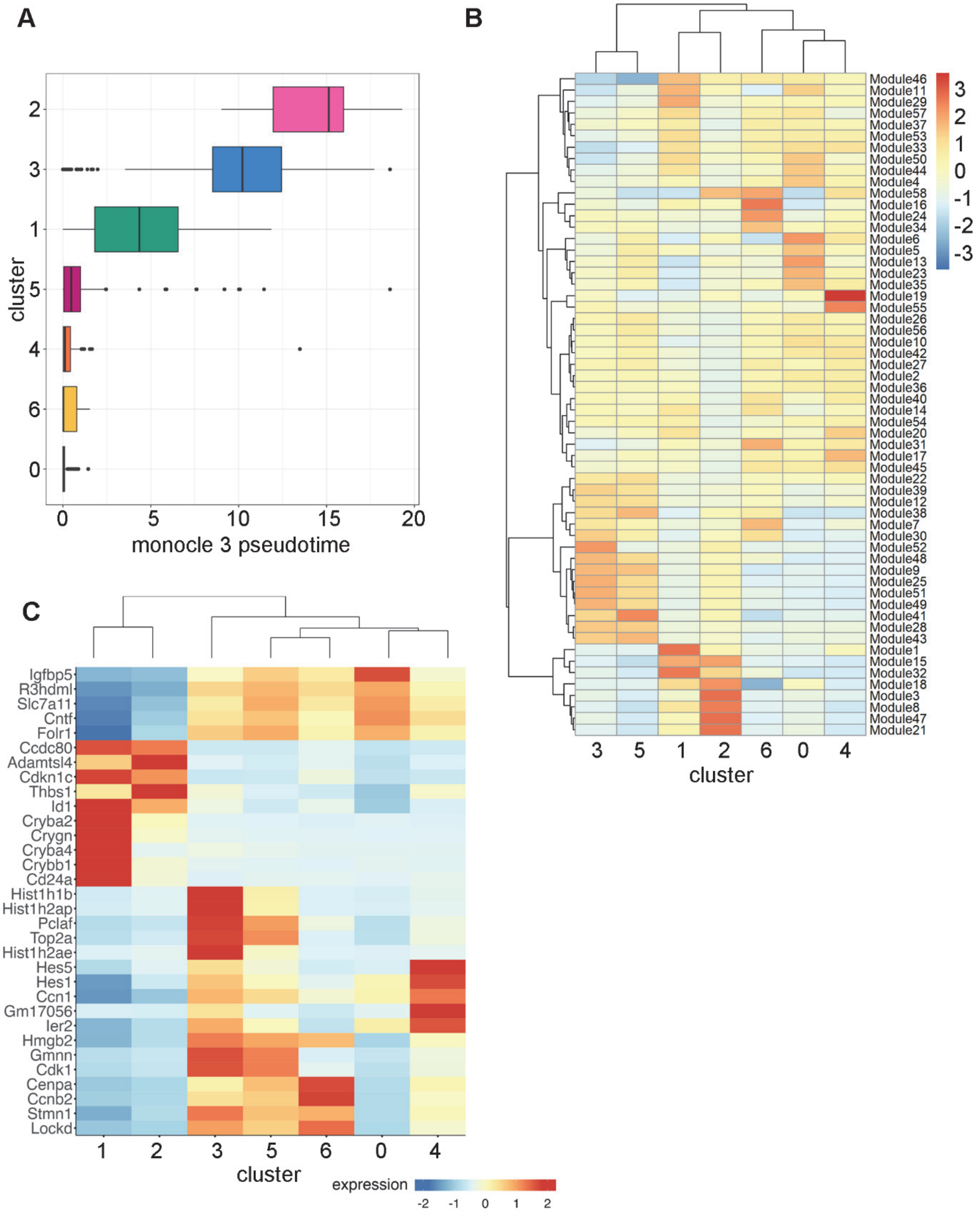

Supplementary Figure 3. (A) Boxplot showing the ordering of clusters by Monocle3 pseudotime scores. (B) Monocle3 heatmap of gene modules for LECs. (C) Heatmap showing hierarchical analysis of clusters.

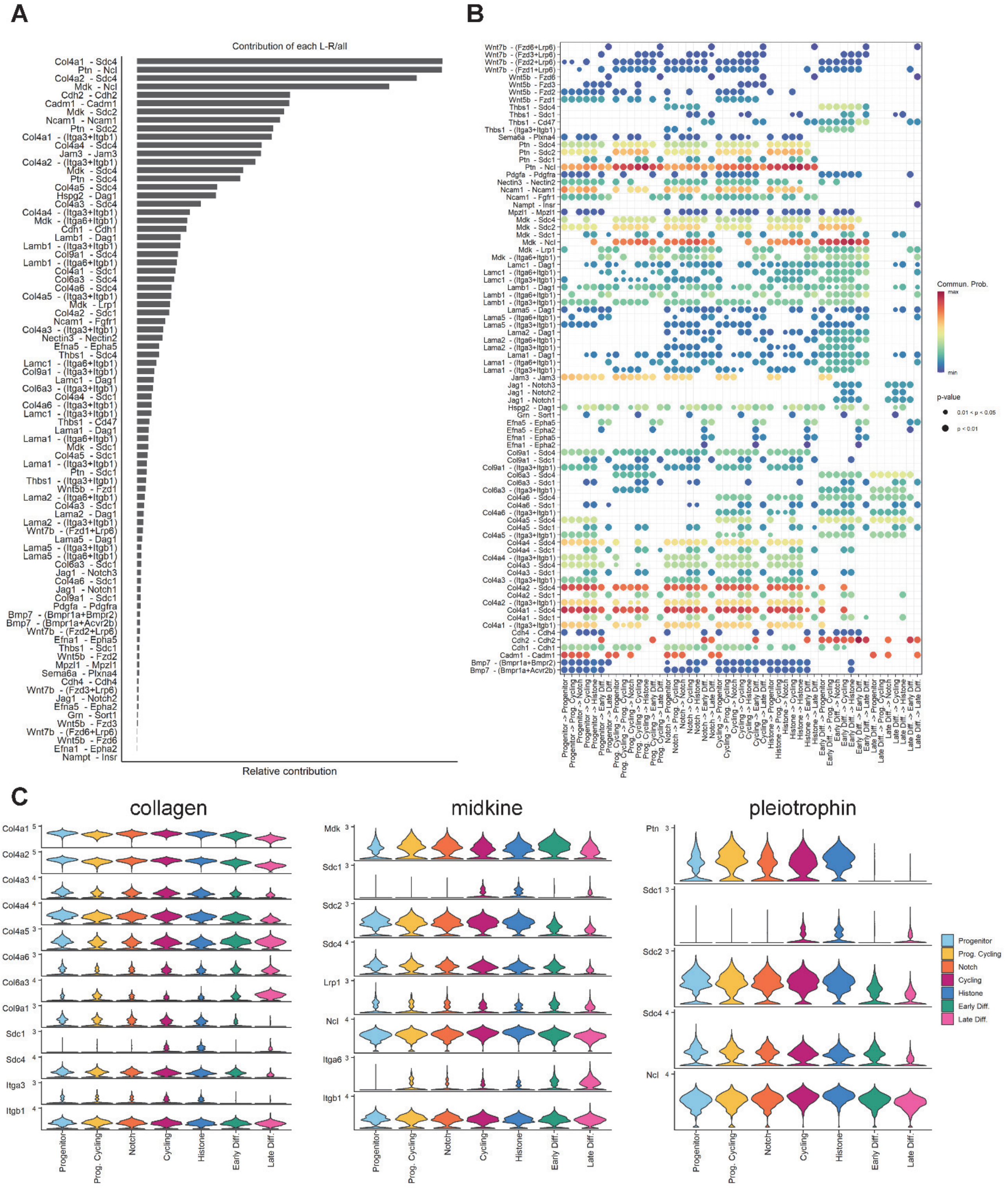

Supplementary Figure 4. (A) Ordering of top ligand-receptor interactions for all LECs by relative contribution. (B) A bubble plot of all significant/identified ligand-receptor interactions between LEC subtypes. (C) Violin plots of expression of genes involved in Collagen (left), Midkine (middle), and Pleiotrophin (right) receptor-ligand interactions.

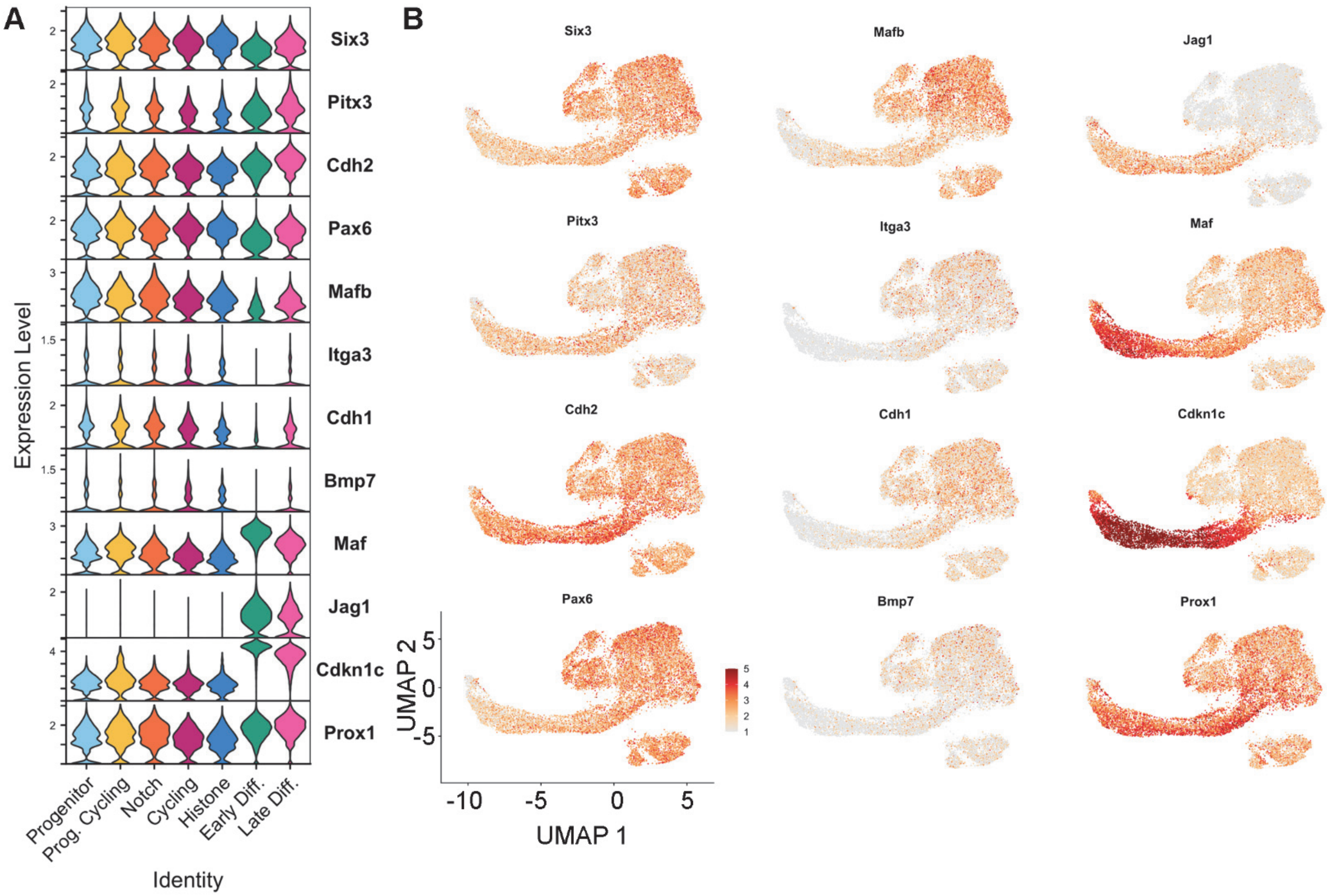

Supplementary Figure 5. (A) Stacked violin plot and (B) feature plots of selected genes with well established roles in LEC homeostasis, growth, and differentiation. Genes were selected from Martinez and de Iongh (2010) DOI: 10.1016/j.biocel.2010.09.012.
